# Design of Tetra-Peptide Ligands of Antibody Fc Regions Using In Silico Combinatorial Library Screening

**DOI:** 10.3390/ph16081170

**Published:** 2023-08-17

**Authors:** Marko Jukič, Sebastjan Kralj, Anja Kolarič, Urban Bren

**Affiliations:** 1 Laboratory of Physical Chemistry and Chemical Thermodynamics, Faculty of Chemistry and Chemical Engineering, University of Maribor, Smetanova ulica 17, SI-2000 Maribor, Slovenia; 2 Faculty of Mathematics, Natural Sciences and Information Technologies, University of Primorska, Glagoljaška ulica 8, SI-6000 Koper, Slovenia; 3 Institute of Environmental Protection and Sensors, Beloruska ulica 7, SI-2000 Maribor, Slovenia

**Keywords:** peptide design, in silico combinatorial library, peptide combinatorial library, peptide library design, high-throughput virtual screening, peptide molecular docking, antibody purification, peptide drug design, recombinant peptide libraries

## Abstract

Peptides, or short chains of amino-acid residues, are becoming increasingly important as active ingredients of drugs and as crucial probes and/or tools in medical, biotechnological, and pharmaceutical research. Situated at the interface between small molecules and larger macromolecular systems, they pose a difficult challenge for computational methods. We report an in silico peptide library generation and prioritization workflow using CmDock for identifying tetrapeptide ligands that bind to Fc regions of antibodies that is analogous to known in vitro recombinant peptide libraries’ display and expression systems. The results of our in silico study are in accordance with existing scientific literature on in vitro peptides that bind to antibody Fc regions. In addition, we postulate an evolving in silico library design workflow that will help circumvent the combinatorial problem of in vitro comprehensive peptide libraries by focusing on peptide subunits that exhibit favorable interaction profiles in initial in silico peptide generation and testing.

## 1. Introduction

Antibodies represent the most widespread molecules in use for analysis and recognition, having reached a 50% market share in diagnostics as early as 1990 [1,2]. Since then, their use has expanded across several fields from bio-recognition agents in molecular biochemistry and diagnostics to biopharmaceuticals [3,4]. Specifically, methods such as enzyme-linked immunosorbent assay (ELISA), immunocytochemistry, flow cytometry, immunoprecipitation, immune-purification in industrial downstream processing, Western blot and biosensors are antibody-based along with certain immunotherapy, immunosuppressant, and infectious disease drugs [1,3,5,6]. Widespread use of antibodies is possible due to their desirable properties, such as a high level of specificity and affinity to target molecules, the ability to target diverse molecules and long shelf-life. [6,7]. Desired antibody properties stem from the mixture of unique structural properties, as they can easily be modified and refined using genetic engineering, with the most notable technology development being the groundbreaking discovery of monoclonal antibodies by Köhler and Milstein, which allowed us to treat antibodies as molecules and not variable biological serum [8]. 

**Figure 1:**
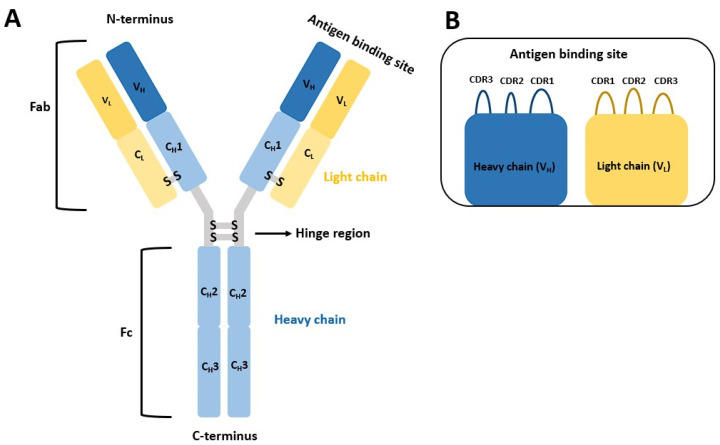
(**A**) The general structure of an antibody resembling the proverbial Y. The smaller antibody domains are denoted. Of special importance are the C_H_3, C_H_2 domain, responsible for binding protein A and the V_L_ and V_H_ domains which both have the highly variable domain. (**B**) The antigen-binding side with the complementarity-determining region (CDR) with all three variable loops that determine antibody specificity.

Antibodies represent heterodimeric proteins, composed of two interconnected heavy and light chains [9,10]. The two arms of the proverbial letter Y represent the variable fragment antigen binding (Fab) domain, responsible for antigen recognition (Figure 1) [10]. The Fab domain contains six hypervariable loops, three from the heavy chain and three from the light chain, dubbed complementarity-determining regions (CDRs). The CDRs’ gene segments, especially encoding for CDR3, are subject to frequent recombination, resulting in antibodies with different antigen specificity [10,11,12]. The less variable Fc domain dictates the immunoglobulin isotype, with five known isotypes (IgM, IgG, IgA, IgD and IgE) present in humans, and is also responsible for interactions with various effector cells (10,13,14,15) (Figure 1) [10,13,14,15]. 

**Figure 2:**
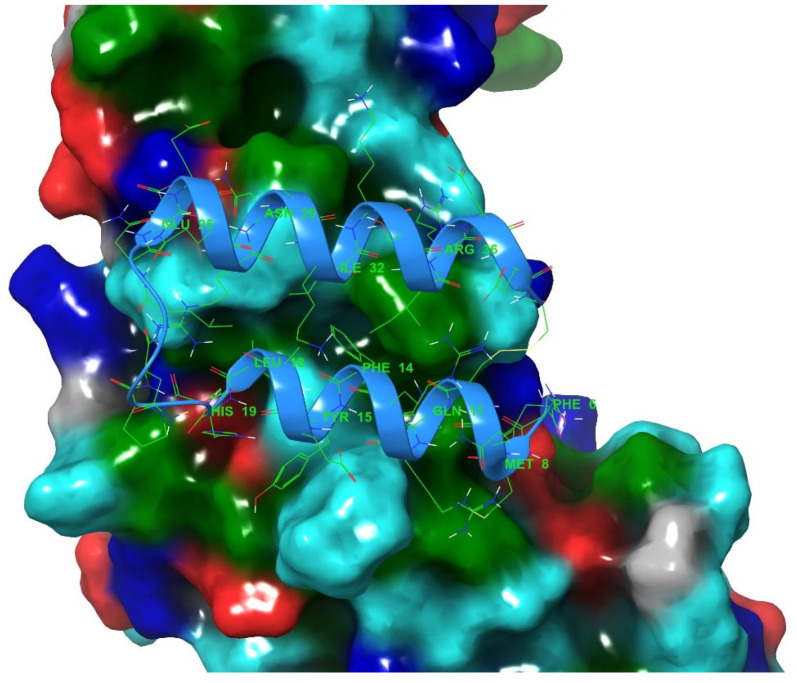
Antibody Fc region (chain A, PDB ID: 5u52) is represented in surface model with bound protein A mini Z domain (chain E) depicted in a cartoon model colored light blue (with residues in green in the line model). Antibody surface is depicted and color-coded according to surface amino-acid residue properties (red and blue indicate negatively and positively charged surface residues, respectively, cyan indicates polar amino-acid residues, green hydrophobic amino-acid residues and gray glycines). Protein A residues in direct contact with the antibody are labelled.

Both antibody domains form interactions with receptors, in turn facilitating the various functions of the antibody [16,17]. The antibodies also represent binding partners for a variety of bacterial proteins, including proteins A, G, L and Z, with the protein A (Figure 2) binding specifically between the CH2 and CH3 domains of the Fc region [16,18]. These interactions are crucial for facilitating the development of antibodies into biological drugs as they are utilized for antibody purification, antibody localization on nanomaterials and for improvement of serum protein half-lives [1].

**Figure 3:**
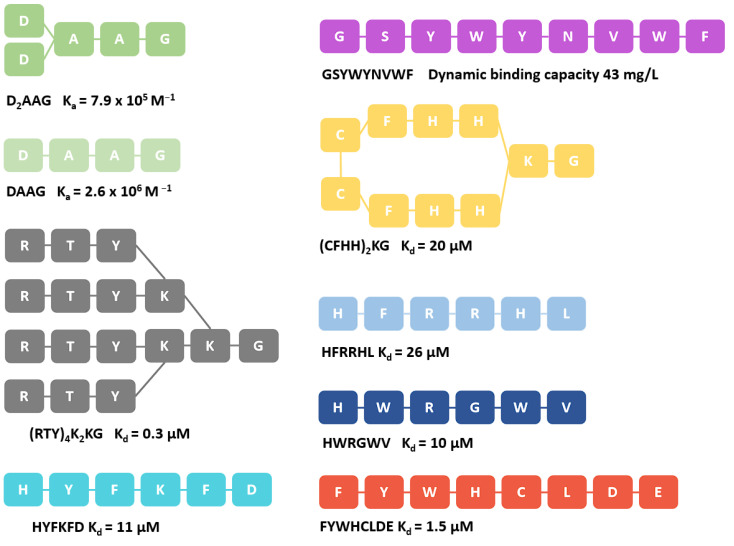
Representative peptides with measured binding affinity. We can observe that the peptide structures vary and can adopt branched, cyclic, and linear patterns [25].

However, as such purification is expensive, amounting to up to 50–80% of the overall production cost, and with the demand for antibodies growing each year, alternative methods with improved properties for the affinity capture of IgG are required. Besides the high production cost, antibodies purified using proteins A and G are burdened by additional factors such as activity loss due to harsh elution or sterilization conditions, as well as leaching of toxic and immunogenic fragments into final products [19,20]. Ligands that would substitute proteins A and G fall short of the logarithmic removal value (LRV) set by protein A, despite an almost three-decade effort to improve on them [20]. Small peptides are currently the most promising candidates to replace proteins A and G as biorecognition reagents [3,4,20]. Despite their small size, usually 4–20 amino-acid residues (AAs), short peptides can bind the target Fc region with specific interactions. Their small size also comes as an advantage because it facilitates cost-efficient synthesis and resistance to harsh elution conditions, since no complex folding patterns are required for biorecognition [3,18]. In comparison with proteins A and G, they also possess low toxicity and immunogenicity profiles [4]. Short peptides capable of binding the Fc domain of antibodies exist in different shapes such as branched, cyclic and linear, of variable length (Figure 3) [21,22,23,24].

Several successful attempts at developing such short peptides exist, such as the hydroxybenzyl-based ligand D2AAG and DAAG, peptides HYFKFD, HDRRHL and HWRGWV, mimetic peptide (RTY)4K2KG, the protein A-based FYWHCLDE and the enriched peptide GSYWYNVWF [3,25,26,27]. The identification of these short peptides has been achieved using high-throughput screening methods, such as phage display libraries or synthetic solid-phase libraries (Figure 3) [3,28]. 

As the importance of computational methods in drug discovery and research is growing, due to their fast workflow, the areas of their applications expand. One such field is peptide–protein interactions which form the key component of all protein–protein interactions (PPI) present in living cells [29]. The initial step in elucidating the important biological processes mediated by peptide segments and ranking them is often achieved using molecular docking [30]. Molecular docking can provide the binding pose of a given peptide, and an assessment of its binding affinity via a scoring function [31]. All in all, peptide docking possesses inherent challenges such as: shallow and solvent-exposed binding sites, larger degrees of freedom compared to small organic molecules of comparable size, binding specificity dependent on key amino-acid residues and secondary structure formation (folding) of peptides [32]. The combination of these factors significantly increases the sampling difficulty for a given docking method and affects the quality of the obtained protein–peptide complexes [32]. Dedicated peptide protocols in docking software such as SP Peptide in the Glide software by Schrödinger are found in manuals [33]. In general, there are also peptide-targeted docking solutions with two prevalent approaches, namely template-based docking and template-free docking, with the former being limited for general applications due to the lack of known templates. The template-free approach can be divided into global docking, in which the binding site is unknown and the docking algorithm searches the entire protein surface, and local docking, in which the docking algorithm searches binding sites within the predefined binding site.

For the benefit of the reader, we collected current peptide-docking approaches in Table 1. The general consensus is that docking algorithms designed specifically for protein–peptide docking outperform protein–protein and small molecule-docking algorithms. However, their performance is heavily dependent on the length of the peptide. For example, typical Glide 2013-2 limits the use to around 11 amino-acid residues or less; the BioLuminate Peptide Docking protocol by Schrödinger to 16 amino-acid residues; Glide 2015-1 and higher can handle up to 100 rotatable bonds and is, therefore, suitable for most peptide cases. As an alternative with regards to the sampling problematics, peptide conformations can be pre-generated using dedicated software and the calculated structures used in rigid docking experiments [30]. 

In this work we, applied a widely parametrized (protein and nucleic acid systems) and an in house developed docking software CmDock that has a feature of extended sampling for the enrichment of a combinatorial tetrapeptide library on the antibody Fc region receptor to develop a useful peptide library docking pipeline that can identify experimentally supported hits. The study performed and the protocol developed may help in vitro researchers in faster discovery of peptide ligands, especially those used in purification processes, potentially realizing the need for a purification peptide with better properties than protein A. As a proof of concept for the in silico protocol, we focused on the smallest peptides showing affinity and selectivity; furthermore, tetrapeptides displayed a manageable sampling problem where the complete combinatorial library of 160,000 tetrapeptides displayed an average of 14.3 ± 2.4 rotatable bonds [25]. The approach of targeting the larger Fc region using small tetrapeptides can also act as a model study for the development of peptides that bind to alternative targets. A small peptide can act as a model ligand, as it has been shown that, despite the involvement of large surfaces in protein–protein interactions, the majority of the energetic contributions for binding comes from key amino-acid residues [34,35]. In this manner, the protein–protein or peptide–protein aspect of the human interactome can be assayed for future drug development as an inverse screening paradigm [36].

**Table 1:** A list of current peptide docking focused software (Accessed on 5 July 2023; servers marked with * were not accessible).

Software Name	Type	Description
pepATTRACT [37]	Global docking	Web server for blind large-scale peptide–protein docking
MDockPeP [38]	Global docking	Protein–peptide docking server, that uses *ab-initio* methods to generate the peptide from sequence and docks it to the receptor structure
GalaxyPepDock [39]	Template-based	A protein–peptide docking tool based on interaction similarity and energy optimization
PIPER-FlexPepDock [40]	Global docking	High-resolution peptide–protein docking using a fragment-based approach, that is founded on the Rosseta fragment picker
CABS-Dock [41]	Global docking	Standalone web server for flexible docking of peptides to proteins without prior knowledge of the binding site
HPEPDOCK [42]	Global docking	A web server for blind protein–peptide docking through a hierarchical algorithm
ClusPro PeptiDock [43]	Global docking	A web server for protein–protein docking with efficient global docking of peptide recognition motifs using fast Fourier transform.
rDock [32]	Local docking	Small molecule docking program, suitable for docking 6–10 amino-acid residue peptides
CmDock	Local docking	A versatile open source fork of the small molecule docking program rDock, suitable for docking various ligands to proteins and nucleic acids
ZDOCK server [44]	Global docking	A protein-docking server for the prediction of protein–protein complex structures and symmetric multimers, based on the rigid-body docking programs ZDOCK and M-ZDOCK
FRODOCK [45]	Global docking	Flexible and fast rotational protein–protein docking
HawkDock [46]	Global docking	A web server * to predict and analyze a given protein–protein complex based on computational docking using the ATTRACT docking algorithm, the HawkRank scoring function and MM/GBSA free energy decomposition for key amino-acid residues.
DINC [47]	Local docking	Auto-dock adapted protocol for docking large ligands
Rosseta FlexPepDock [48]	Global docking	An ab initio approach to simultaneous folding, docking and refinement of peptides onto their receptors
AutoDock CrankPep [49]	Local docking	Flexible peptide docking to rigid receptors based on folding and docking
PeptoGrid for AutoDock [50]	Local docking	Rescoring function for AutoDock based on frequency information of ligand atoms
DynaDock [51]	Local docking	Molecular dynamics based algorithm for flexible protein–peptide docking
GOLD [52]	Local docking	Docking software based on genetic algorithm for flexible ligand docking
Surflex [53]	Local docking	Flexible molecular docking software based on a molecular similarity-based search engine

## 2. Results

### 2.1. Library Preparation

**Figure 4:**
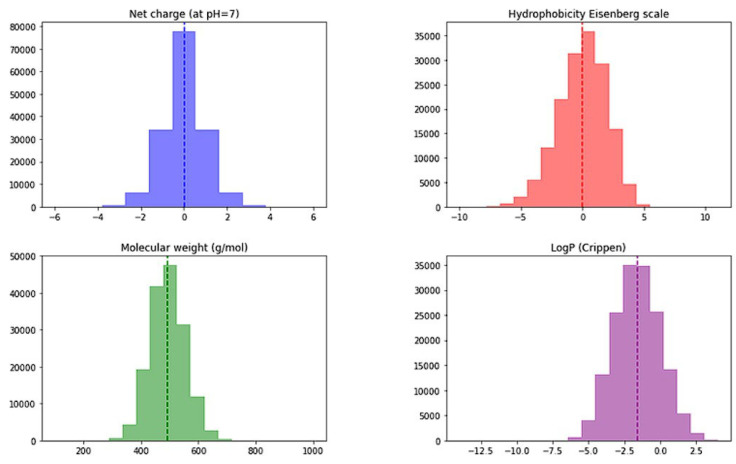
Histograms of net charge (average net charge based on pka values of each amino acid at pH = 7), molecular weight (average calculated in g/mol using the SMILES representation), Crippen logP (estimation of the octanol/water partition coefficient using the Ghose/Crippen approach available in the RDKit project) and Eisenberg’s hydrophobicity (calculated by averaging the values of each amino acid hydrophobicity value from the Eisenberg scale) for the generated 160 k tetrapeptide library.

Peptide library preparation was performed using Python PepFun libraray [54]. Initially, a complete combinatorial library of tetrapeptides consisting of ACDEFGHIKLMNPQRSTVWY amino-acid residues was constructed consisting of 160,000 tetrapepides. Frequency analysis of Ala at all four positions afforded 5% (interestingly PepFun library offers also ‘natural’ library generation where Ala frequency analysis for position 1 is: 5.42, for position 2: 6.6, for position 3: 3.75 and 5.42% for position 4). We calculated sequence properties of the obtained combinatorial library, namely net charge (the average is 0.006), molecular weight (average of 493.6 g/mol), Crippen logP (average −1.608) and Eisenberg’s hydrophobicity (average of 0) and the resulting histogram plots are presented in Figure 4.

The initial conformer generation (pdb output) using PepFun was performed and supplemented with Yasara Structure to obtain a complete 3D structural set for the combinatorial library of 160k tetrapeptides [55]. Yasara peptides were modelled residue-by-residue, cleaned, optimized using the “SCWRL3” method and saved as pdbs. The method optimizes side chains using a rotamer library developed by Canutescu et al. [56]. Lastly, a docking sdf library was prepared using the GNU Parallel, Open Babel toolbox [57] and ionized using the OpenEye QUACPAC (OpenEye Scientific Software. Santa Fe, NM, USA; www.eyesopen.com, accessed on 5 July 2023) to obtain ionization states at pH 7.4. [58,59]. Tasks were parallelized with the help of Gnu Parallel by Ole Tange [59]. The library was docked in the next step using CmDock (v. 0.2.0; https://gitlab.com/Jukic/cmdock; accessed on 5 July 2023) [60].

### 2.2. Binding Mode Analysis

We present the results of our study and analyze the consensus where the 100 highest-scored tetrapeptide ligands were selected for further chemical space investigation (the top 10 tetrapeptides were also selected for reference). Binding poses of ligands were visually examined and clustered into five clusters based on their binding mode (using interaction fingerprints as implemented in Maestro by Schrödinger SMD, Release 2022-1, Schrödinger, LLC, New York, NY, 2022). Eleven out of 100 tetrapeptides have alternative binding poses, therefore, they could not be classified into any cluster and represent outliers (presented in Supporting information). Within each cluster we have examined which interactions dictate ligand binding and which amino-acid residues are the most vital at which tetrapeptide position to ensure the binding. 

**Figure 5:**
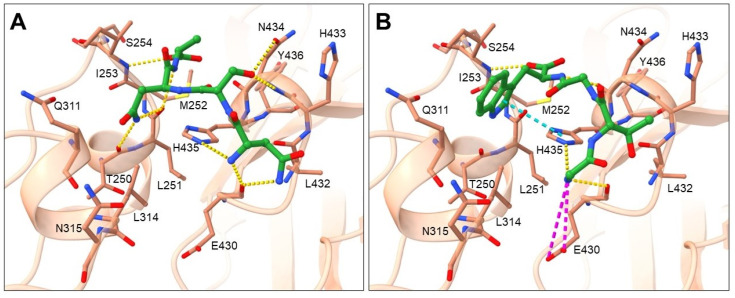
Binding mode of cluster 1 NSNA (**A**) and GTGW (**B**) representatives reflecting different conformations within the cluster. The ligands are shown in green stick representation, while the Fc region of the antibody is depicted by a light brown cartoon showing the major interaction amino-acid residues in stick representation. Salt bridge interactions are denoted with magenta, π-π with cyan, and H-bonds with yellow dashes.

Cluster 1 represents the biggest cluster and contains 46 tetrapeptides, the binding mode of which is presented in Figure 5 by two of the highest ranked ligands NSNA (Figure 5A) and GTGW (Figure 5B).

Amino-acid residues from the Fc region of the IgG antibody that showed the highest number of interactions with tetrapeptide ligand are shown in Figure S2 (Supporting information) and include hydrogen bond acceptor residues: THR250, LEU251, GLU430, LEU432 and HIS435; hydrogen bond donor residues: ILE253 and ASN434; hydrophobic residues: MET252, ILE253 and LEU314; and a charged residue GLU430. According to ligand binding modes and frequency of amino-acid residues occurrence, small differences in binding mode can be observed within the cluster. Amino-acid residues most frequent at peptide position 1 can be placed into two groups. The first group contains amino-acid residues with uncharged side chains (SER, THR, ASN, GLN, CYS) that donate hydrogen atoms into a hydrogen bond with the carbonyl of GLU430 main chain (Figure 5A). In this conformation, the *N*-end forms H-bonds with carbonyl of GLU430 main chain and imidazole nitrogen of HIS435 side chain and in certain cases also with carbonyl of LEU432 main chain. In the second group are the smallest residues, GLY and ALA, in which the *N*-end is shifted slightly toward the GLU430 side chain, which facilitates additional interaction, namely with GLU430 carboxylate (Figure 5B). Amino-acid residues having uncharged side chains, such as SER, THR or ASN, are present at peptide position 2 that engage in H-bond interaction with carbonyls of GLU430 or LEU432 main chains or amine/amide of ASN434. Amino-acid residues at peptide position 3 include residues able to donate hydrogen, such as positively charged HIS, uncharged ASN, THR, and CYS, as well as residues with aromatic hydrophobic side chains (PHE, TRP). The pattern that was observed was that if residues with aromatic cyclic side chain (HIS, PHE or TRP) and ASN are present at position 3, they are oriented toward the binding pocket formed by the Fc residues LEU251, MET252, ILE253, GLN311, and LEU315, where they interact through H-bonding with a carbonyl of the THR250 main chain and aromatic cycle containing residues HIS, TRP, and PHE form π-π stacking interactions with HIS435 (Figure 5A). Due to the high hydrophobicity of side chains at this position, the largest share of ligand hydrophobic interactions was observed with residues MET252, ILE253 and LEU314 as seen from Figure S2 (Supporting information). In cases when amino-acid residues with smaller side chains as THR or CYS and in some cases also ASN are at position 3, they are oriented toward ASN434 and form an H-bond with its main or side chain. Similarly, amino-acid residues with hydrogen bond-donating side chains, such as positively charged ARG and HIS, uncharged ASN, HIS and SER, as well as residues with aromatic hydrophobic side chains (PHE, TYR, and TRP), are the most common residues at position 4. When residues with smaller side chains (THR, CYS, GLY and in some cases also ASN) are present at position 3, that cannot occupy the binding pocket formed by the Fc residues LEU251, MET252, ILE253, GLN311, and LEU315, then it is noticeable that this binding pocket is occupied with the most optimal residues ASN, HIS, PHE, and TRP at position 4, which form an H-bond with carbonyl of the THR250 main chain and aromatic cycle containing residues HIS and TRP, which form π-π stacking interactions with HIS435 (Figure 5B). When the pocket is occupied with residues at position 3, the amino-acid residues found at position 4 are the ones with smaller side chains (Figure 5A). The main interaction at the C-end represent H-bonding with the amine of the ILE253 main chain. The ligand backbone forms an H-bond mainly with imidazole nitrogen of HIS435 side chain, amine of ASN434, carbonyl of LEU251 and carbonyl of GLU430 main chains. Although the amino-acid residues binding at the first two positions were similar for all the ligands, some deviated from the presented binding pattern at residues 3 and 4. These include ligands SRKS, CNYA, TNSY, ANKK, AWQN, CCKR and QTNC. 

**Figure 6:**
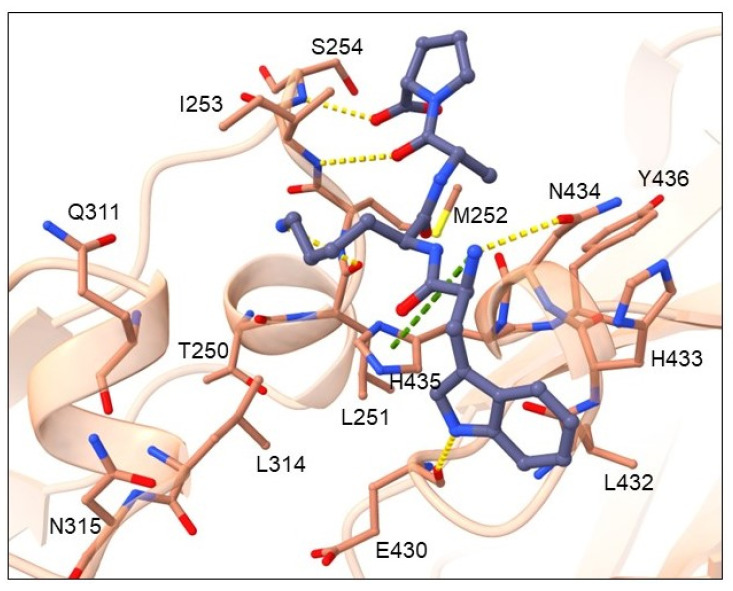
Binding mode of cluster 2 WKAP representative. The ligand is shown in dark blue ball-and-stick representation, while the Fc region of the antibody is depicted by a light brown cartoon displaying the major interaction amino-acid residues in ball-and-stick representation. Cation-p interactions are denoted with green and H-bonds with yellow dashes.

Cluster 2 contains 16 tetrapeptides and their general binding mode is presented in Figure 6 by the highest-ranked ligand WKAP.

Amino-acid residues from the Fc region of IgG antibody that showed the highest number of interactions with the tetrapeptide ligand are shown in Figure S3 (Supporting information) and include hydrogen bond acceptor residues: THR250, GLU430, and ASN434; hydrogen bond donor residues: ILE253, SER254 and ASN434; hydrophobic residues: MET252, ILE253, LEU314, and TYR436; and an aromatic residue TYR436. According to ligand-binding modes, the most common amino-acid residues at peptide position 1 are TRP or ARG, which donate H into a hydrogen bond with the carbonyl of GLU430 main chain. Residues with a smaller, OH-containing side chain at position 1, such as THR, form an H-bond with main and side chains of ASN434. Residues with H-bond donating side chains, such as positively charged HIS, ARG, and LYS, as well as hydrophobic, aromatic cycle containing TRP at position 2, form an H-bond with the carbonyl of the THR250 main chain in the binding pocket formed by Fc residues LEU251, MET252, ILE253, GLN311, and LEU315. These ligand side chains are also involved in hydrophobic interactions with residues MET252, ILE253 and LEU314. These are the same residues as identified in the binding pocket of cluster 1. Prominent interactions of residues at position 3 (such as LYS and HIS) are π-π or π-cation interactions with TYR436. Otherwise, positions 3 and 4 contain residues GLY, PRO, and ALA. The *N*-end forms an H-bond with the amide of the ASN434 side chain and π-cation interaction with the imidazole of the HIS435 side chain. The C-end interacts with the amine of the SER254 side chain through an H-bond. The ligand backbone forms an H-bond with the ILE253 backbone nitrogen (Figure 6). 

**Figure 7:**
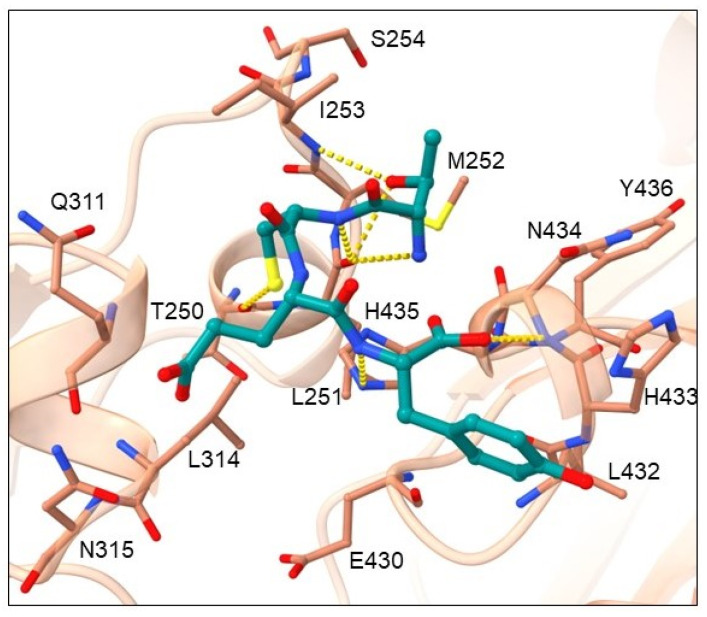
Binding mode of cluster 3 TCEY representative. The ligand is shown in dark green ball-and-stick representation, while the Fc region of the antibody is depicted by a light brown cartoon displaying the major interaction residues in ball-and-stick representation. H-bonds are denoted with yellow dashes.

Cluster 3 contains nine tetrapeptides and their binding mode is presented in Figure 7 by the highest-ranked ligand TCEY.

Amino-acid residues from the Fc region of IgG antibody that showed the highest number of interactions with the tetrapeptide ligand are shown in Figure S4 (Supporting information) and include hydrogen bond acceptor residues: LEU251, GLN311, ASN434 and HIS435; hydrogen bond donor residues: ILE253, and ASN434; as well as hydrophobic residues: MET252, ILE253, and LEU314. Amino-acid residues that are the most favorable at peptide position 1 contain OH side chains (THR and SER), which form H-bonds with the carbonyl of the LEU251 main chain and/or the amine of the ILE253 side chain. Residues at peptide position 2 (CYS, LYS, and HIS with uncharged and charged side chains, respectively) donate H into an H-bond with the carbonyl of the THR250 main chain. These residues are in hydrophobic contact with Fc amino-acid residues MET252, ILE253 and LEU314. The most common residues at position 3 represent residues LYS and GLU with charged side chains and SER with an uncharged side chain, while the most common residue at position 4 is TYR with a hydrophobic side chain. Common to all these residues is their ability to donate a hydrogen atom into an H-bond with Fc residues GLN311, ASN315 or GLU430. The *N*-end forms an H-bond with carbonyls of the LEU251 and/or ASN434 main chains. The C-end conformation is very variable, but most interactions could be identified with the amine of ASN434 main chain. The ligand backbone mainly engages in H-bonding with the carbonyl of the LEU251 main chain and/or the imidazole nitrogen of the HIS435 side chain. 

**Figure 8:**
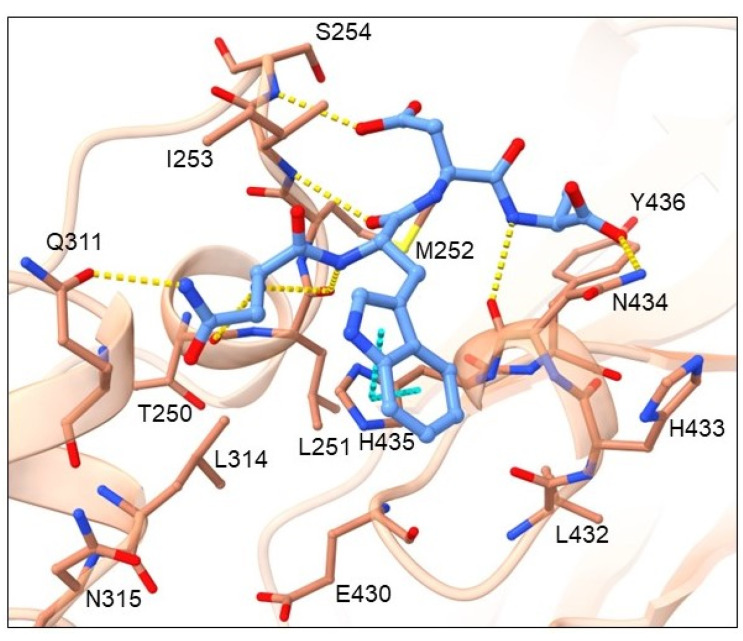
Binding mode of cluster 4 NWDA representative. The ligand is shown in light blue ball-and-stick representation, while the Fc region of the antibody is depicted by a light brown cartoon displaying the major interaction residues in ball-and-stick representation. π-π interactions are denoted with cyan and H-bonds with yellow dashes.

Cluster 4 contains 11 tetrapeptides and their binding mode is presented in Figure 8 by the highest-ranked ligand NWDA.

Amino-acid residues from the Fc region of IgG antibody that showed the highest number of interactions with the tetrapeptide ligand are shown in Figure S5 (Supporting information) and include hydrogen bond acceptor residues: THR250, LEU251, GLU430, and ASN434; hydrogen bond donor residues: ILE253, SER254 and ASN434; hydrophobic residues: MET252, ILE253, LEU314, and TYR436; as well as aromatic residue, TYR436. CYS and ASN at peptide position 1 donate H into an H-bond with the amide of the GLN311 main chain. Residue position 1 also contributes the most to hydrophobic contacts with Fc amino-acid residues MET252, ILE253 and LEU314. The most common residue at peptide position 2 contains an aromatic system R-group that forms π-π stacking interactions with the imidazole of the HIS435. Position 2 can also be occupied by ARG that forms an H-bond with the carbonyls of the LEU432 and/or GLU430 main chains. The *N*-end forms an H-bond with the carbonyls of the THR250 and/or LEU251 main chains. The main interactions of the ligand backbone include H-bonds with the carbonyls of LEU251 and ASN434 as well as the amine of the ILE253 main chains. The C-end can enter a conformation in which it forms an H-bond with the amine of the SER254 main chain or a conformation in which it forms an H-bond with the amide of the ASN434 side chain. The tetrapeptide backbone contacts the carbonyl of LEU251 and ASN434 and the amine of the ILE253 main chains through H-bonding. 

**Figure 9:**
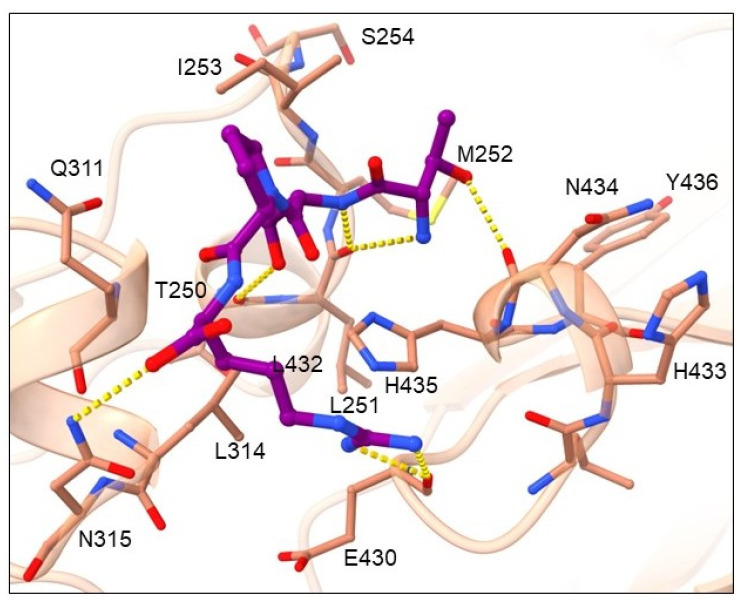
Binding mode of cluster 5 TSPR representative. The ligand is shown in violet ball-and-stick representation, while the Fc region of the antibody is depicted by a light brown cartoon displaying the major interaction residues in ball-and-stick representation. H-bonds are denoted with yellow dashes.

Cluster 5 is the smallest and contains seven tetrapeptides, and their binding mode is presented in Figure 9 by the highest-ranked ligand TSPR.

Amino acid residues from the Fc region of IgG antibody that showed the highest number of interactions with tetrapeptide ligand are displayed in Figure S6 (Supporting information) and include hydrogen bond acceptor residues: THR250, LEU251, GLU430, LEU432 and ASN434; hydrogen bond donor residues: ASN315, and ASN434; as well as hydrophobic residues: MET252, ILE253, and LEU314. According to ligand-binding modes, residues at position 1 contain smaller side chains with the OH group (SER and THR) that donate hydrogens into H-bonds with the carbonyl of the ASN434 backbone. Hydrogen-donating residues with polar uncharged side chains (SER, THR, ASN) are also present at position 2 and form H-bonding interactions with the carbonyl of the THR250 side chain. Simultaneously, they are in hydrophobic contact with the Fc amino-acid residues MET252, ILE253 and LEU314. When ARG is present at position 2, it rotates and occupies the binding pocket of residues at position 3 in which it forms an H-bond with the carbonyl of the GLU430 main chain. Amino-acid residues at position 3 contain an aromatic system R-group (TYR and HIS) that forms H-bonds with the carbonyl of LEU432 or the amine of the ASN434 main chain. The *N*-end forms an H-bond with the carbonyls of LEU251 and/or the ASN434 main chain. The C-end interacts with the amide of the ASN315 side chain.

## 3. Discussion

**Figure 10:**
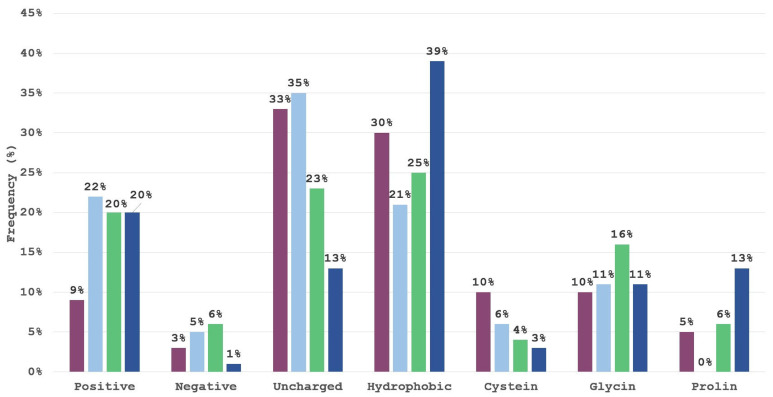
Frequency of certain amino acid groups by tetrapeptide position for the top 100 scoring ligands. Blue represents the first position, gray second, green third and orange the fourth position.

One of the patterns observed is that residues ARG, LYS, HIS, ASN or THR represent the most suitable residues to occupy the binding pocket formed by Fc residues LEU251, MET252, ILE253, GLN311, and LEU315, while in cluster 4, the *N*-end enters this binding pocket. However, to further dissect the preference of amino acids for individual positions within the tetrapeptide, and to complement the results obtained by visual inspection, we examined the frequency of AAs for the top 100 highest-scoring tetrapeptides. Using in-house KNIME workflows, we classified AAs according to their side-chain properties. The selected groups were positive (R,H,K), negative (D,E), polar uncharged (S,T,N,Q), hydrophobic (A, V, I, L, M, F, Y, W), cysteine (C), glycine (G) and proline (P). Each of the four positions in the tetrapeptide was examined individually. The results are visualized in Figure 10.

The first position in the tetrapeptide is dominated by interactions of the uncharged polar and hydrophobic groups, with the two together representing a total of 63% of all interactions. The majority of top-scoring ligands possess uncharged polar amino acids in the second position of the tetrapeptide, with positive and hydrophobic amino acids; coming in second, cysteine and glycine as small amino acids also form large groups. The results seem to indicate that a positively charged amino acid is not favored for the first tetrapeptide position and that negatively charged amino acids are under-represented. The third position in the tetrapeptide is the most diverse with the positive, uncharged polar and hydrophobic group having a frequency of at least 20%. The fourth position, however, is largely dominated by hydrophobic amino acids and positively charged amino acids, which are present in the first cluster, and include residues with aromatic hydrophobic side chains (PHE, TYR, TRP) as well as the positively charged amino acids ARG and HIS. Proline is present in the fourth position in the tetrapeptide of the second cluster and has a frequency of 13% but is generally unfavored in this system.

**Figure 11:**
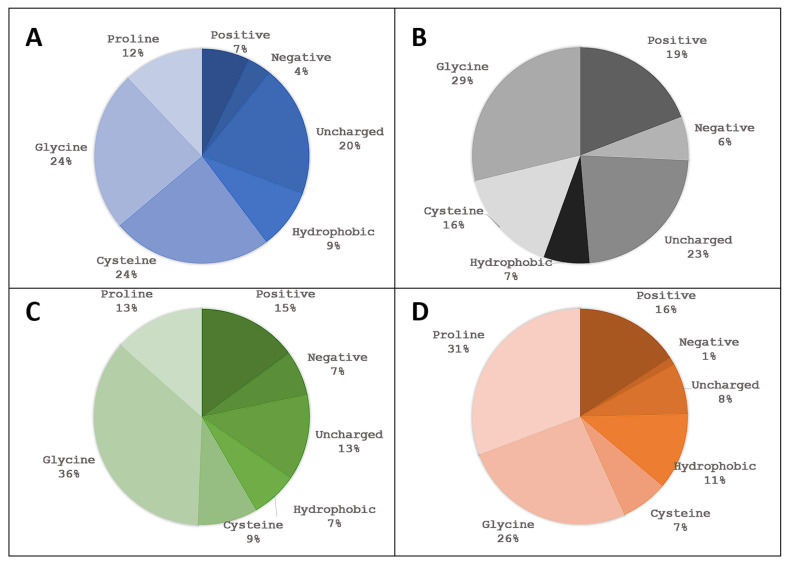
Normalized frequency of amino acid groups by individual tetrapeptide position. Blue (**A**) represents the first position, black/grey (**B**) the second, green (**C**) the third and orange (**D**) the fourth position.

Since the size of classification groups differs, we normalized the results according to the classification cluster size and the normalized frequencies are presented below in Figure 11.

Normalized results expectedly demonstrate that a significant proportion of top-scoring ligands exhibit a high prevalence for hydrophobic or uncharged polar residues at position 1, hydrophobic, uncharged polar or positive residues at position 2, and hydrophobic residues at positions 3 and 4 in accordance with observations made by Bratkovič et al. when performing phage display selections [3]. Despite the negatively charged amino acid group consisting of two amino acids only they are very poorly represented even in the normalized results, however their involvement could be expected, if tetrapeptides would be evolved to cover a larger protein A-contacting surface. Glycine residue is also to be expected as it allows for a greater conformational flexibility and helps in a favorable positioning of neighboring residues.

**Figure 12:**
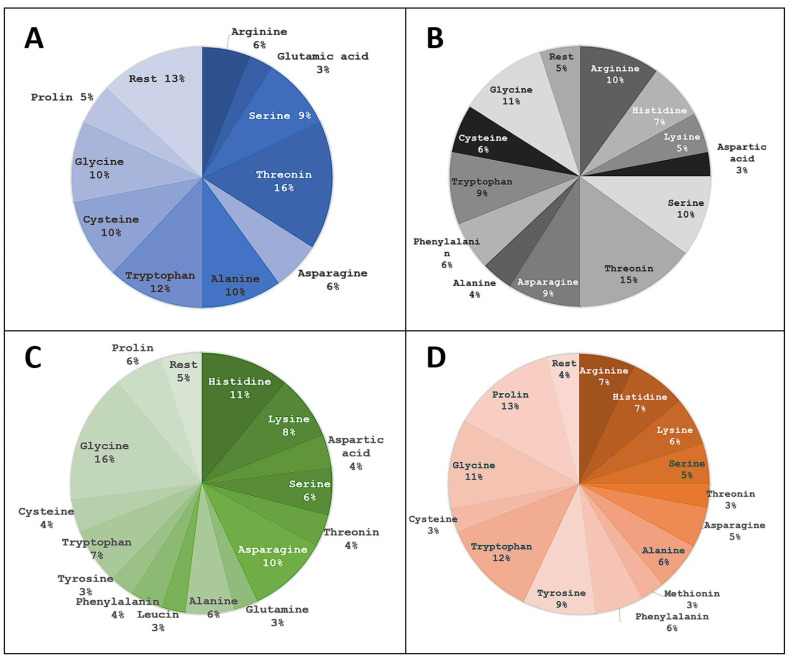
Frequency of amino-acid residues by tetrapeptide position. Blue (**A**) represents the first position, black/grey (**B**) the second, green (**C**) the third and orange (**D**) the fourth position. AAs present in less than two ligands were summed and represent the rest group of the pie chart.

Moreover, to dig into the individual classifications, we analyzed individual amino acids at each of the four positions (Figure 12) in the top 100 ranked tetrapeptides.

Similar to the results obtained with classification groups, we see that positions 1 and 2 are less diverse. In particular, the first position has six amino-acids with their frequency over 10%. An example is threonine, which has a frequency of 16% at the first position and a frequency of 15% at the second position. In the third position, the fairly large part consists of glycine and, in the fourth position, tryptophan and tyrosine residues (proline should be analyzed individually as it often displays unique binding modes and singleton clusters).

**Figure 13:**
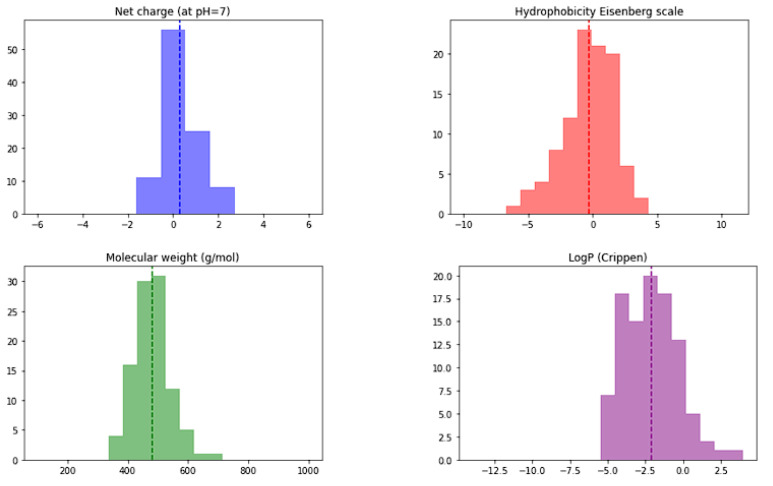
Histograms of net charge (the average of 0.313; net charge based on pka values of each amino acid at pH = 7), molecular weight (the average of 481.7 g/mol), Crippen logP (average −2.120; estimation of the octanol/water partition coefficient using the Ghose/Crippen approach available in the RDKit project) and Eisenberg’s hydrophobicity (the average of −0.311; calculated by averaging the values of each amino acid hydrophobicity value from the Eisenberg scale) for the top 100 ranked tetrapeptides.

The initial descriptor set was analyzed to see if there are significant differences between all tetrapeptides present in the combinatorial library versus the top 100 scoring tetrapeptides. The initial set of library descriptor histograms is presented in Figure 13.

**Figure 14:**
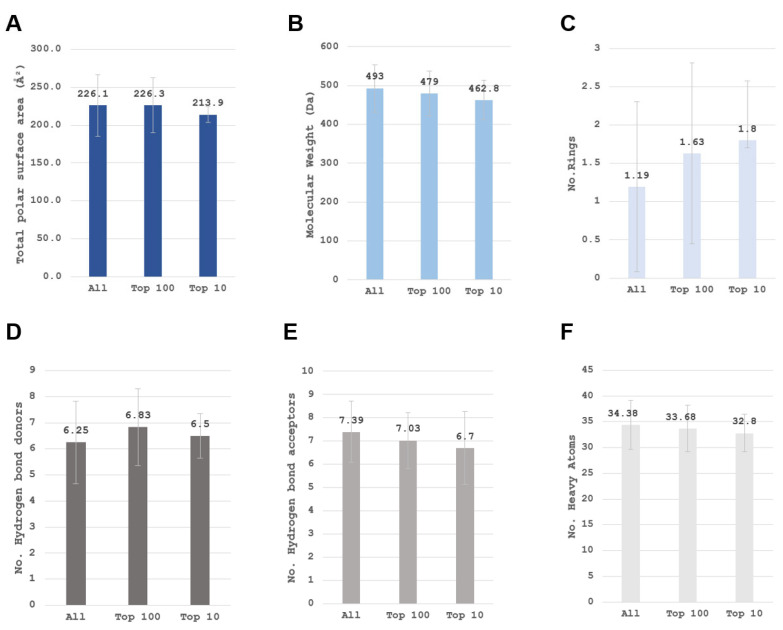
Comparison of basic descriptors for the entire tetrapeptide library and the top 100 as well as top 10 scoring ligands. (**A**) Total polar surface area; (**B**) Molecular weight reference; (**C**) Number of rings (rings present in proline, histidine, phenylalanine, tyrosine, tryptophan); (**D**) Hydrogen bond donors; (**E**) Hydrogen bond acceptors; (**F**) Number of heavy atoms.

Mean descriptor values of the top 100 ranked peptides are similar to the initial combinatorial library reflecting the variety of amino acids in top-scoring peptides; however, histogram skewness hints at a higher representation of larger logP and generally hydrophobic tetrapeptides in the identified enrichment set. In order to elaborate on this observation, an additional set of basic small-molecule descriptors is depicted in Figure 14. The means of top scorers do not differ significantly from the generated combinatorial library within the examined descriptor set. The major observed difference is the larger number of rings (rings present in histidine, proline, tyrosine, tryptophan and phenylalanine) and the expected lowering of polar surface area due to the positions 2 and 4. As discussed before, position 1 is predominantly occupied by uncharged residues with the ability to donate hydrogen bonds. A slight trend of lower molecular weight and number of heavy atoms is also present with the top 10 scorers pointing to lower molecular weights and a larger number of hydrogen atoms relative to heavy atoms present for these ligands. This allows the atoms to form a greater number of hydrogen bonds, a fact backed up by the larger number of hydrogen donors found for both the top 100 and top 10.

The general descriptor set mean similarities point to the fact that the spatial orientation or the unique sequence position of amino acids within the tetrapeptide are more important than general physio-chemical properties when it comes to binding affinity. This observation could help in developing future peptide drug design knowledge-based molecular filters, where one should employ a peptide-specific and position-oriented filtering pipeline instead of employing general a small-molecule-oriented filtering pipeline as is often seen in the scientific literature [56].

**Figure 15:**
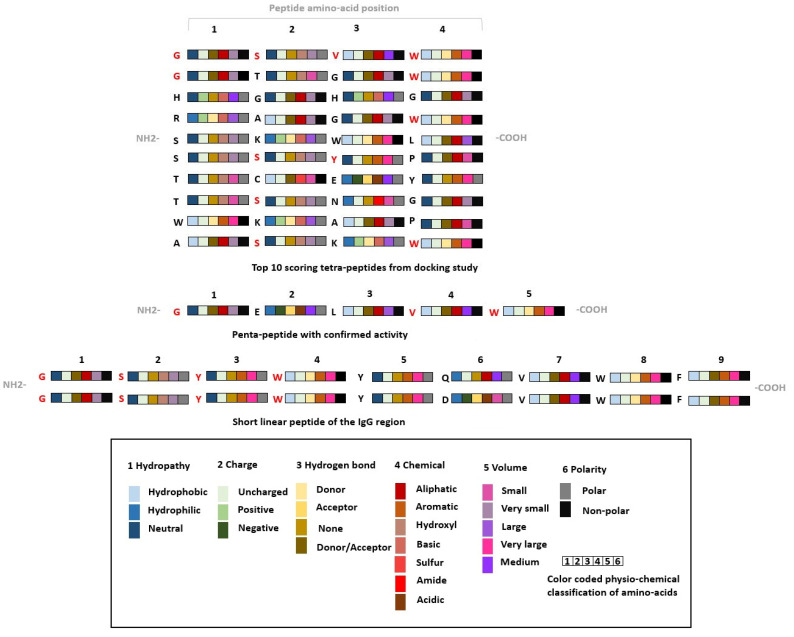
Amino-acid sidechain property analysis of our top-scoring tetrapeptides [3,61].

The CmDock identified preferred amino-acid positions of the top-scoring tetrapeptides are in accordance with the ones reported by the work of Bratkovič et al. and observations made by DeLano et al. [3,61]. The peptide ligand GSYWYQVWF identified from a combinatorial phage-display library screen possesses glycine at the first position, an amino-acid residue with an uncharged side chain that can donate hydrogen atoms into a hydrogen bond. Serine at the second position acts as a small uncharged amino-acid residue capable of engaging in hydrogen-bond interactions. Tyrosine and tryptophan at the third and fourth position, both aromatic amino acids, are also present in cluster 1 and responsible for forming interactions with the described Fc region binding pocket. Our identified cluster 1 incorporates similar motifs to the identified peptides by Bratkovič et al. and the cluster contains some of the highest-scoring tetrapeptides [3]. In an additional comparison of our results with the tetrapeptide docking study performed by Fang et al. [62], we again observe the presence of larger hydrophobic residues at multiple positions (identified tetrapeptide YEHF); however, comparison cannot be fully made due to selection of AAs from six “critical” residues made by Fang et al. (Phe, Tyr, Leu, Glu, Ile, Lys) with one additional histidine incorporated for experimental peptide purification. The similarity of our top tetrapeptide hit GSVW and common amino-acid motifs in top 10 scorers is presented in Figure 15.

**Figure 16:**
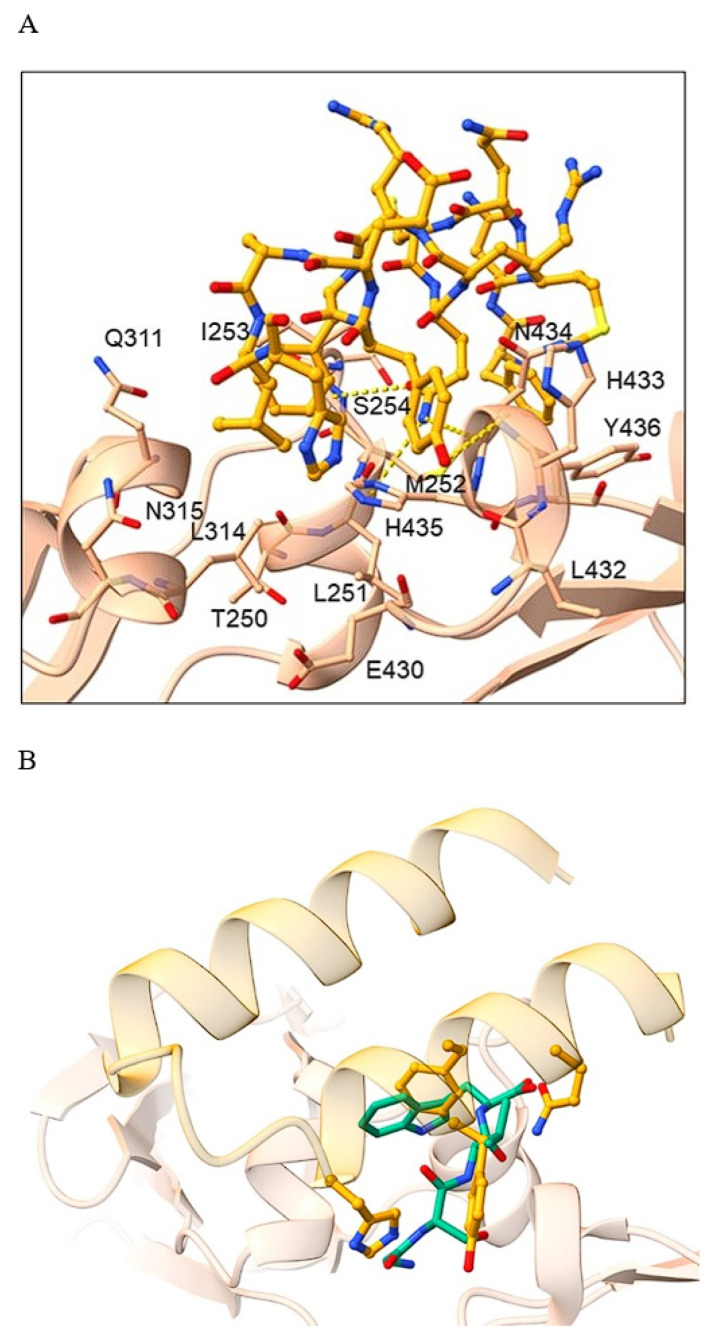
(**A**): Binding mode of protein A. The protein A is shown in orange ball-and-stick representation, while the Fc region of the antibody is denoted by a light brown cartoon depicting the major interaction residues in ball-and-stick representation. H-bonds are delineated with yellow dashes. (**B**): Superposition of protein A mini Z domain binding mode is presented in yellow cartoon and emphasizes the binding of amino-acid residues in ball-and-stick representation with the top-scoring tetrapeptide GSVW in green stick representation. An analogous positioning of peptide Trp4 to the mini Z domain Phe14 can be observed.

Structural comparison of the identified tetrapeptide binding modes with the binding mode of protein A mini Z domain is depicted in Figure 16 and Figure S1 (Supporting information).

We can observe that our identified top hit peptides can effectively mimic the binding of the protein A mini Z domain to the Fc region of the antibody. In Figure 16, the top-scoring tetrapeptide GSVW can be seen with its *N-end* at the His residue of the protein A mini Z domain, S effectively supplementing the Tyr OH group and a two-amino-acid hydrophobic end with analogous positioning of tetrapeptide Trp 4 into the binding pocket for the protein A mini Z domain Phe 14 residue (position 9 of the mini Z domain sequence: FNMQCQRRFYEALH DPNL NEEQRNAKIKSIRDDC with helix-turn-helix secondary structure). We observed that our peptide compounds can extend toward TYR436 and form mainly hydrophobic interactions, therefore prolonging the tetrapeptide into a pentapeptide, which could also lead us to target TYR436 and elaborate all subsequent positions. Attaching aromatic (PHE, TYR, TRP) or charged (ARG, HIS, LYS) residues at position 5 could be beneficial for the formation of π-π and cation-π interactions, respectively, which effectively corresponds to the experimentally observed structures and validates our protocol for potential applications on alternative targets. 

## 4. Materials and Methods

Molecular docking was performed using CmDock (v. 0.2.0; https://gitlab.com/Jukic/cmdock; accessed on 5 July 2023) [60]. The goal was to prioritize tetrapeptides and enrich the generated library towards tetrapeptides capable of forming meaningful interactions with the Fc region of the antibody. CmDock is an in-house developed open-source docking software fitted for extended sampling (via number of GA runs parameter) and its current CPU implementation is ideally suitable for parallel deployment on HPC resources [60]. We used the experimental crystallized antibody IgG1 Fc system (PDB ID: 5U52; excellent resolution of 1.94 Å) with co-crystallized 2 helix minimized version of the B-domain from protein A ligand (Mini Z domain; K_D_ value of mini Z domain was determined at 25 nM using surface plasmon resonance) and prepared the system applying chains A and E (according to temperature factors) with Yasara Structure software [55]. We defined the docking grid of 4 Å around the large reference ligand heavy atoms using following parameters: RbtLigandSiteMapper, radius 4.0, small_sphere 1.0, min_volume 100, max_cavities 1, vol_incr 0.0, and gridstep 0.5. Explicit water molecules were not considered during molecular docking. With 100 runs utilizing DOCK.prm settings, we applied a sample technique that included three stages of genetic algorithm search, low-temperature Monte Carlo, and simplex minimization phases, as well as scoring using the rDOCK (SF3) scoring function [31]. We employed 100 runs as a comprehensive sampling parameter where suitable docking calculation times can be achieved with converging average docking scores. However, upon average INTER.SCORE convergence analysis on random 0.001 samples, we also advocate the use of larger-run ensembles, especially if longer calculation times per peptide are feasible and larger peptides are being studied. The sampling and scoring were validated against protein [31,63] and RNA [31,64] targets, and their performance was superior to similar open-source software [32]. We applied the receptor flexibility parameter (RECEPTOR_FLEX 3.0) to activate sampling of terminal OH and NH_3_+ groups in the 3 Å vicinity of the docking site. The docking results were investigated using PyMOL, in-house developed scripts, and Konstanz information miner (KNIME) software [65].

## 5. Conclusions

We performed a high-throughput virtual screening campaign on the Fc region of an IgG antibody using CmDock and an unbiased combinatorial tetrapeptide library. We compared the consensus of obtained data with the experimental results of Bratkovič et al. [14]. We were able to validate the proposed protocol and identify similar amino-acid motifs as beforehand reported experimentally. Our top-scored tetrapeptide GSVW motif can be clearly identified in the GSYWYQVWF peptide by Bratkovič et al. and further peptide evolution using docking studies with even greater sampling populations in CmDock should be able to address the design of larger peptides where additional protein-folding problems occur. We firmly believe that this approach represents a useful tool for future peptide elongation studies. Specifically, we aim at employing enriched hit-libraries of smaller peptides to be evolved, rather than employing outright combinatorial libraries of final-length peptides as demonstrated by this work. Research on small peptides can afford valuable probes and/or leads to design peptide mimetics, or one can use their pharmacophoric profiles to identify novel drug-like moieties that can utilize identified interactions [66]. In addition, their synthesizability potential places them firmly as affinity research tools to explore novel binding sites or modes of action.

## Data Availability

Data is contained within the article and Supplementary Material.

## Supplementary Materials

The following supporting information can be downloaded at: https://www.mdpi.com/article/10.3390/ph16081170/s1, Figure S1: Showing a representative compound with its binding pose for each of 5 clusters. (A) NSNA and GTGW (in green), representatives of cluster 1. (B) WKAP (in dark blue), representative of cluster 2. (C) TCEY (in dark green), representative of cluster 3. (D) NWDA (in light blue), representative of cluster 4. (E) TSPR (in violet), representative of cluster 5. Ligands are shown in ball and stick representation, while the Fc region of the antibody is represented as light brown surface.; Figures S2–S6: Interaction fingerprints of hydrogen bond acceptor; Figure S3: Interaction fingerprints of hydrogen bond acceptor (A) hydrogen bond donor (B) and hydrophobic residues (B), showing the residues that showed maximum number of interactions with peptide ligands in cluster 1 to 6, respectively.

## Abbreviations

HTVS—high-throughput virtual screening, CDRs—complementarity-determining regions, Fc—fragment crystallizable region.
