# Supplementary material for: Design of Tetra-Peptide Ligands of Antibody Fc Regions Using In Silico Combinatorial Library Screening
Source: Pharmaceuticals (Basel). 2023 Aug 17;16(8):1170. doi: 10.3390/ph16081170 (PMC10459493; doi:10.3390/ph16081170)
Supplement: Supplementary file 1 [file pharmaceuticals-16-01170-s001.zip › pharmaceuticals-2552272-supplementary.pdf]

pharmaceuticals

Supporting Information

for

Design of Tetra-peptide Ligands of Antibody Fc Regions Using *In Silico* Combinatorial Library Screening

Marko Jukič[1,2,3,*], Sebastjan Kralj[1], Anja Kolarič[1,3] and Urban Bren[1,2,3*]

[1]Laboratory of Physical Chemistry and Chemical Thermodynamics, Faculty of Chemistry and Chemical Engineering, University of Maribor, Smetanova ulica 17, SI-2000 Maribor, Slovenia
[2]Faculty of Mathematics, Natural Sciences and Information Technologies, University of Primorska, Glagoljaška ulica 8, SI-6000 Koper, Slovenia
[3]Institute of Environmental Protection and Sensors, Beloruska ulica 7, SI-2000 Maribor, Slovenia

Citation: To be added by editorial staff during production.

Academic Editor: Firstname Lastname

Received: date
Revised: date
Accepted: date
Published: date

Copyright: © 2023 by the authors. Submitted for possible open access publication under the terms and conditions of the Creative Commons Attribution (CC BY) license (https://creativecommons.org/licenses/by/4.0/).

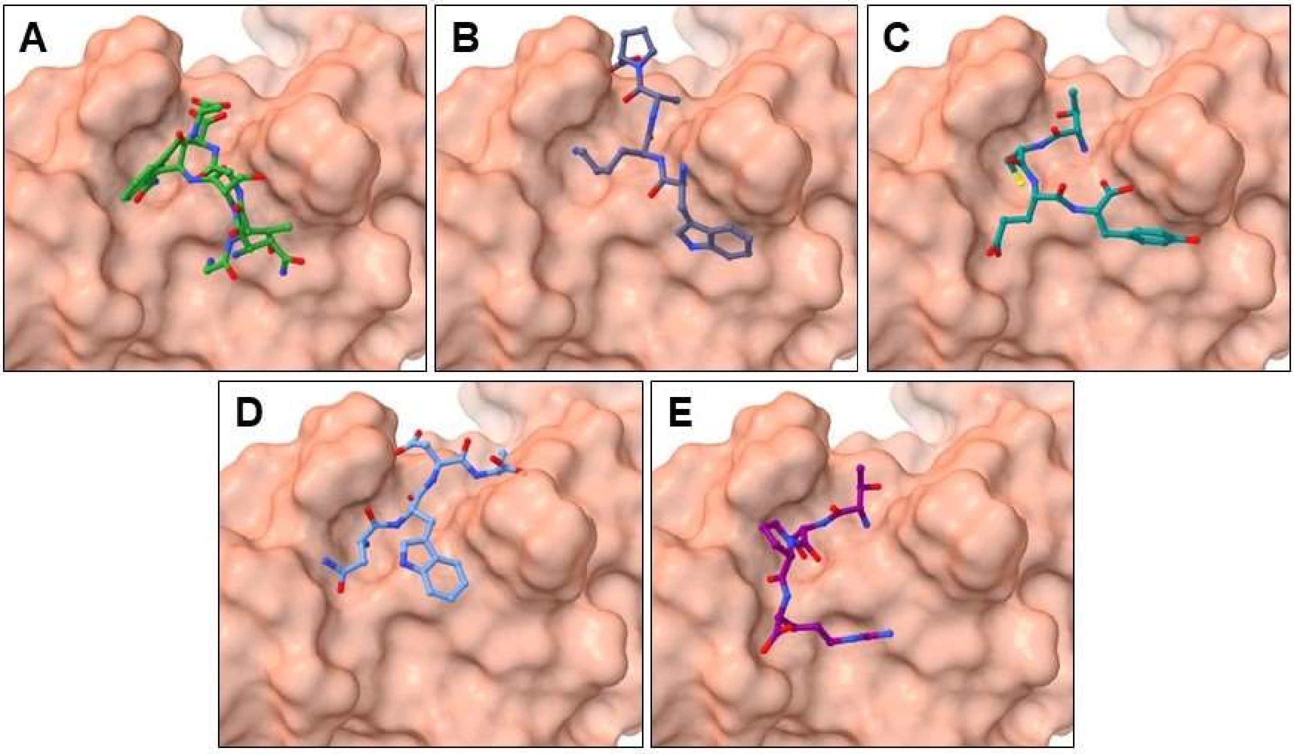

Figure S1. Showing a representative compound with its binding pose for each of 5 clusters. (**A**) NSNA and GTGW (in green), representatives of cluster 1. (**B**) WKAP (in dark blue), representative of cluster 2. (**C**) TCEY (in dark green), representative of cluster 3. (**D**) NWDA (in light blue), representative of cluster 4. (**E**) TSPR (in violet), representative of cluster 5. Ligands are shown in ball and stick representation, while the Fc region of the antibody is represented as light brown surface.

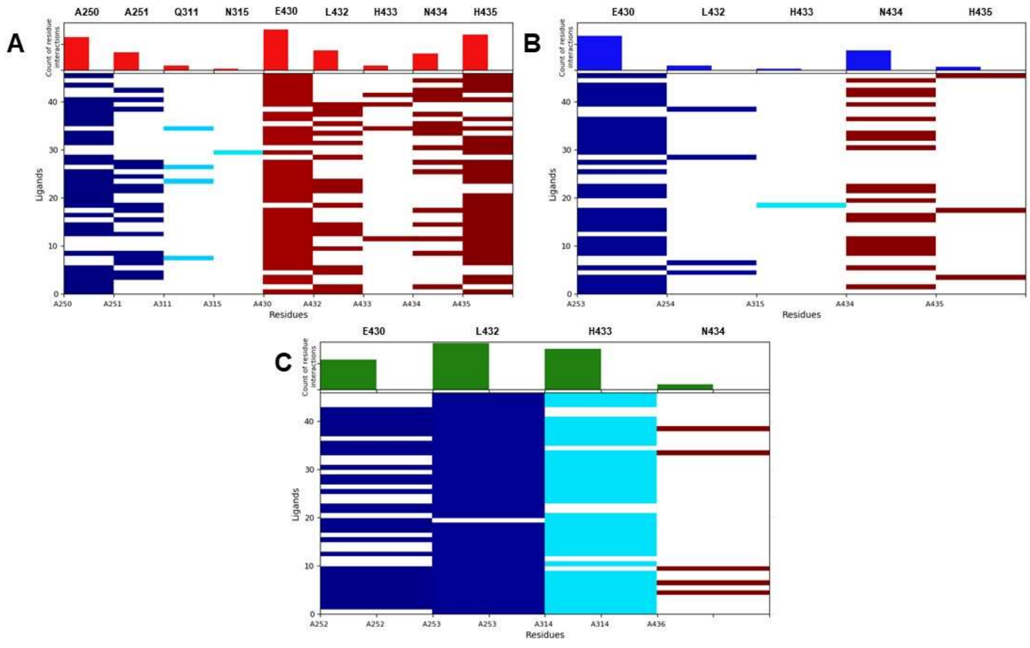

Figure S2. Interaction fingerprints of hydrogen bond acceptor (**A**) hydrogen bond donor (**B**) and hydrophobic residues (**C**), showing the residues that showed maximum number of interactions with peptide ligands in cluster 1.

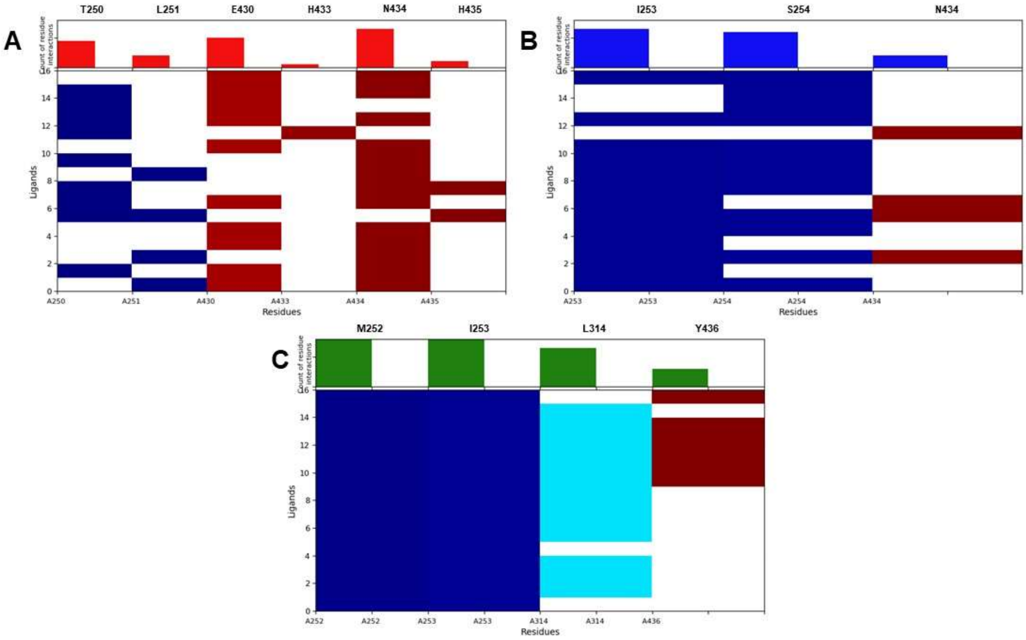

Figure S3. Interaction fingerprints of hydrogen bond acceptor (**A**) hydrogen bond donor (**B**) and hydrophobic residues (**C**), showing the residues that showed maximum number of interactions with peptide ligands in cluster 2.

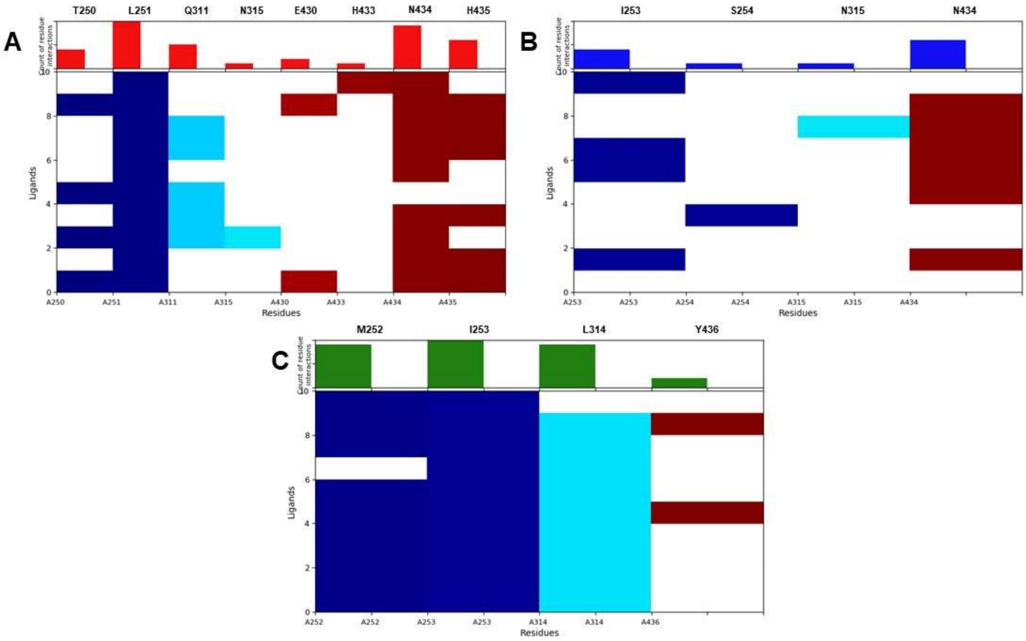

Figure S4. Interaction fingerprints of hydrogen bond acceptor (**A**) hydrogen bond donor (**B**) and hydrophobic residues (**C**), showing the residues that showed maximum number of interactions with peptide ligands in cluster 3.

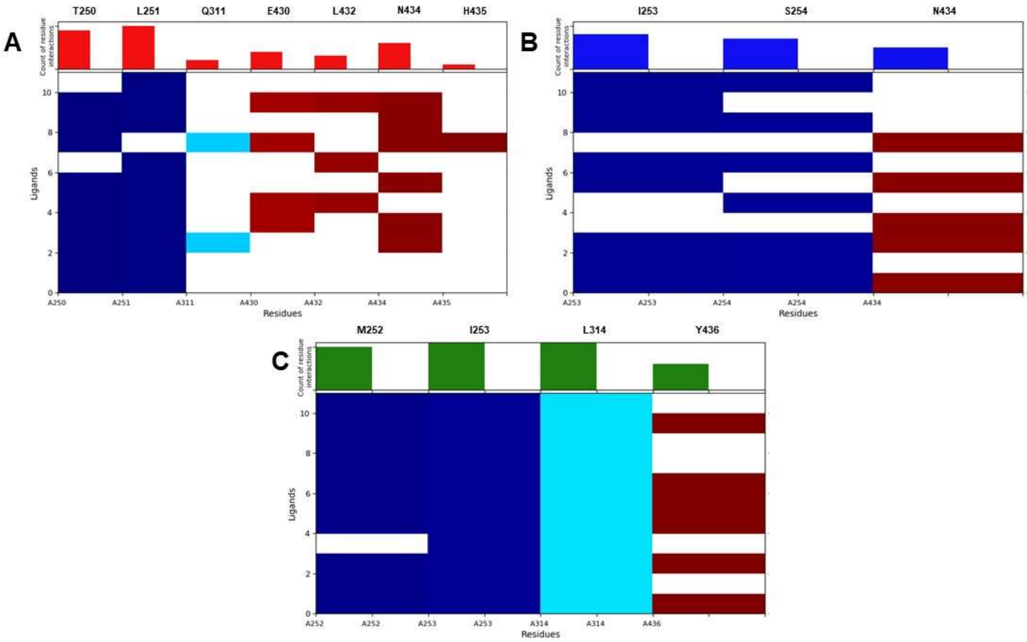

Figure S5. Interaction fingerprints of hydrogen bond acceptor (**A**) hydrogen bond donor (**B**) and hydrophobic residues (**C**), showing the residues that showed maximum number of interactions with peptide ligands in cluster 4.

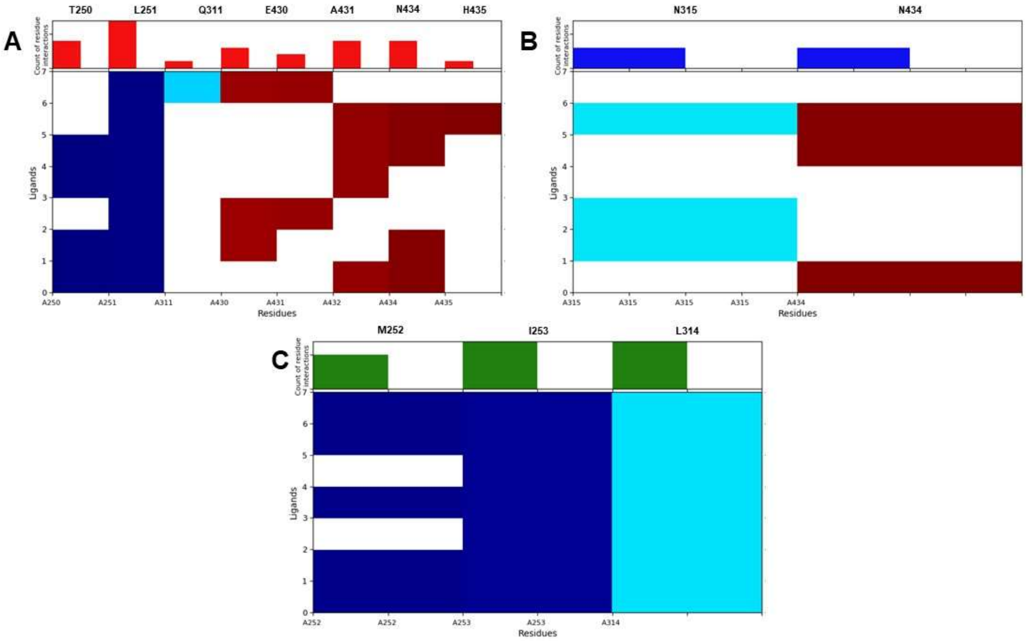

Figure S6. Interaction fingerprints of hydrogen bond acceptor (**A**) hydrogen bond donor (**B**) and hydrophobic residues (C), showing the residues that showed maximum number of interactions with peptide ligands in cluster 5.
